# Point-of-care haemoglobin testing in African hospitals: a neglected essential diagnostic test

**DOI:** 10.1111/bjh.17431

**Published:** 2021-05-15

**Authors:** Sophie Uyoga, Elizabeth C. George, Imelda Bates, Peter Olupot-Olupot, Yami Chimalizeni, Elizabeth M. Molyneux, Kathryn Maitland

**Affiliations:** 1 Kenya Medical Research Institute (KEMRI), Wellcome Trust Research Programme, Kilifi, Kenya; 2 Medical Research Council Clinical Trials Unit (MRC CTU) at University College, London; 3 Liverpool School of Tropical Medicine, Liverpool UK, Liverpool, UK; 4 Faculty of Health Sciences, Busitema University, Mbale Regional Referral Hospital, Mbale, Uganda; 5 College of Medicine, Malawi-Liverpool-Wellcome Research Programme, Blantyre, Malawi; 6 Department of Infectious Disease and Institute of Global Health and Innovation, Division of Medicine, Imperial College, London, UK

**Keywords:** anaemia in developing world, diagnostic haematology, transfusion medicine, paediatric anaemia, children

## Abstract

Owing to the rapid turnaround time in the assessment of haemoglobin level by point-of-care tests (POC Hb), these have grown in popularity and scope in large parts of the world. However, whilst POC testing for malaria and HIV remains has been integrated into patient management in Africa, the use of POC haemoglobin testing remains neglected by health services. The main users of transfusions (paediatric, maternity and trauma services) present largely as emergencies. Ward-based POC Hb could result in more rapid and accurate diagnosis of anaemia, contributing to saving of lives and at the same time reduce unnecessary transfusions which deplete the limited supplies of donated blood in Africa. Severe anaemia requiring transfusion is a major cause of paediatric admission in Africa. At a dissemination meeting to discuss the results of a large phase III paediatric transfusion trial and steps to implementation of the findings participants strongly recommended that one of the most pressing actions required was to prioritise the use of POC haemoglobin testing. This would facilitate implementation of the new transfusion algorithm, developed at the meeting, which refines patient management including blood transfusions. We present the rationale for the strongly recommended prioritisation of POC Hb, using paediatric transfusion as an exemplar.

In many countries in sub-Saharan Africa, in stark contrast to other areas of the world, children are the main recipients of blood transfusions, where severe anaemia [haemoglobin (Hb) <60 g/l] remains a leading cause of both admission to hospital and of direct mortality.^1–4^ Owing to limited supplies of donated blood, the World Health Organization (WHO) guidelines encourage restrictive transfusion approaches. Specifically, these conservative strategies recommend not transfusing children with uncomplicated anaemia (haemoglobin of 40–60 g/l).^5^ However, the evidence informing these recommendations was previously weak and thus adherence was poor. Delays in the provision of an emergency transfusion in children are common, with early fatalities occurring in children awaiting a transfusion.^6,7^

In 2019, a large multicentre phase III trial (TRACT) enrolling 3 983 children with severe anaemia in four hospitals in Uganda and Malawi provided high-quality evidence to support and refine current treatment guidelines.^8,9^ Within the trial, 1 565 children with uncomplicated severe anaemia (haemoglobin 40–60 g/l and no severity features) were randomised to immediate (experimental) *versus* no immediate transfusions (control: WHO standard of care). In this stratum an initial (for control) or additional transfusions were permitted if prompted or ‘triggered’ by the development of new severity signs or post-randomisation haemoglobin <40 g/l. Overall, the TRACT trial found no evidence of differences in 28-day mortality, 180-day mortality or readmissions between immediate and triggered transfusion strategies. Nevertheless, for children in the control arm who followed WHO guidelines (no immediate transfusion), 49% went on to develop severe and complicated anaemia — a clinical scenario that the current guideline does not anticipate. Among those developing severe criteria, in 76% this was diagnosed as a subsequent measurement of haemoglobin that was below 40 g/l (profound anaemia), and in 15% it was due to the development of a new clinical severity sign. As a result this ‘triggered’ the requirement for a blood transfusion. Whilst this was possible in a clinical trial that included frequent monitoring of haemoglobin, in reality this relatively simple task remains an insurmountable problem since point-of-care (POC) diagnostics and access to round-the-clock laboratory services to provide blood counts are lacking in most settings where these children are cared for.

Nevertheless, if this potential barrier for blood transfusions services would be addressed, and if the results of the trial were adopted, a triggered transfusion strategy would reduce blood requirements in the group of children with uncomplicated severe anaemia by ~60% compared to immediate transfusion, thus preserving blood transfusion supplies for emergencies. This finding is important in settings like those where the TRACT trial was conducted, where shortages of donor blood are still frequent,^10^ despite a decade or more of increased external aid to support blood transfusion services.^11^ Moreover, a triggered transfusion strategy is considerably less costly for the health services than immediate transfusions despite the longer length of stay in hospital (around 24 h, on average).^8^ Overall, 28-day mortality (<2%) was substantially lower than expected to which a number of factors may have contributed, including closer clinical surveillance and repeated haemoglobin monitoring, so that those developing severe and complicated anaemia were rapidly identified and provided with transfusions.

Translating the results into clinical practice first needs a careful consideration of potential barriers to implementation and what might be needed to support safe implementation of the TRACT findings.

## Dissemination meeting

A meeting in Kampala, Uganda in February 2020 that was co-hosted by the African Society for Blood Transfusion (AfSBT) and the Uganda Paediatric Association brought together representatives from across the relevant regions in sub-Saharan Africa: blood transfusion services, health service providers (paediatricians involved in providing continued professional development and training courses) and representatives from international policymakers (Médecins Sans Frontières and the Ugandan representative for WHO). The aims were to clarify what current practices are in different settings within Africa, to explore the findings of the TRACT trial and implications from their own perspectives, to identify potential barriers to implementing the TRACT findings, and what might be needed to support safe implementation of the TRACT findings. Finally, the last aim was to develop a package of interventions (which might include training tools, job aids and revised processes) for subsequent implementation research. Here we specifically report on the aspect that was agreed to be a major barrier to effective implementation: timely access to initial and repeated haemoglobin testing in children with suspected severe anaemia.

## Current practice

Prior to the meeting we sent out a structured survey enquiring about local guidelines for management of severe anaemia and including two specific questions on haemoglobin testing. Most clinicians reported that their current guidelines and practice aim to follow WHO guidelines. These guidelines include recommendations that restrict routine transfusion to only those with a haemoglobin less than 40 g/l (haematocrit <12%) or 40–50 g/l if they have additional signs of severity.^5^ Nevertheless, the evidence base for the paediatric guidelines is weak, which has led to confusing recommendations within these guidelines and poor adherence.^7,12^ However, the guidelines give no specific guidance on clinical or haematological post-admission monitoring of children to identify those children who subsequently develop new severity criteria warranting transfusion.

We asked about the most common method used for anaemia diagnosis [POC Hb, haematocrit or full blood count (haemogram)] and how long it generally took to get a haemoglobin/haematocrit (or haemogram) result. Most indicated it would take between 1 and 8 h and 10% indicated the following day. Haemoglobin testing was only reliably available in just under 40% of those facilities. A recent publication on transfusion practice in Kenyan hospitals showed that, after adjusting for such features as severe illness, children for whom a transfusion was ordered and given on the same day had lower mortality [Odds Ratio (OR) = 0·58, 95% confidence interval (CI) = 0·38–0·87] than those whose transfusion was delayed (≥ 1 day after prescription).^6^ Children in whom transfusion was ordered but was never received had a substantially higher in-hospital mortality: 20% vs 12% in those receiving blood (OR = 1·8, 95% CI = 1·3–2·49).^6^ Although there may have been other reasons for the worse outcome in those not receiving a transfusion in this study, we have previously shown that many severely anaemic children (<50 g/l) that could not be transfused in the first 8 h (owing to stocks out at the blood bank) a significant proportion died [54/103 (52%)]; 90% of these deaths occurred within 2·5 h of Hb measurement and 100% had died within 5 h of this timepoint.^7^ We therefore conclude that delays in receipt of blood for transfusion are costing lives in those that need it most. The paediatricians involved in national guideline training who attended the meeting were concerned that adherence to the new algorithm would be difficult unless there is a greater focus on getting POC Hb tests onto the hospital wards, as has been done for malaria and HIV, but not for anaemia.

In the TRACT trial, point-of-care (POC) Hb was available to test any child with suspected severe anaemia (severe pallor), largely taken at hospital admission. The time between screening, enrolment and receipt of the first transfusion was on average 1 h,^8^ which may have accounted for the overall low mortality rate. One other factor that could explain the lower mortality was suspending trial recruitment when donor blood was not available. Despite the strengthening of hospital laboratories, there is substantial evidence that many prescriptions of blood for paediatric transfusion are not supported by a Hb test.^13^ It has been reported that up to 45–65% of paediatric transfusion requests were not based on the result of a Hb reading but on the clinical judgement of the treating physician.^6,12^ In this regard, the clinical indicator clinicians frequently use to guide management is the presence of severe pallor. However, this has been shown to have a low specificity for accurately identifying severe anaemia (Hb < 50 g/l).^14^ In a subanalysis of a trial recruiting children with impaired perfusion, 381/501 (77%) of children with Hb 50–70 g/l (moderate anaemia) and 167/843 (20%) who had Hb 70–100 g/l (mild anaemia) had clinician-recorded severe pallor on clinical examination.^7^ Thus, the clinical utility of pallor as a screening test for suspected anaemia therefore seems more relevant to community assessment and pre-referral.^15,16^

## Point-of-care haemoglobin testing

Despite the recognition by WHO that cheap and reliable measures of haemoglobin are an important tool for health services, such assays have not been prioritised in countries where there is the greatest need.^17^ For example, those designed for other parts of the world have lower limits of detection at Hb ~70–80 g/l.^18,19^ Although a relatively small number have been developed and evaluated for low resource settings^20–23^ in reality none have become more widely utilized or adopted. Moreover, some are only optimised for reading Hb >40 g/l^20,21^ or have been repurposed in experimental research conditions but are currently not available on the market.^23^ EKF Diagnostics (Cardiff, UK) have recently released the DiaSpect TM, a handheld device with a measurement range of 0–255 g/l, with a Bluetooth interface which is yet to be tested in children with severe anaemia (https://www.ekfdiagnostics.com/diaspect.html).

## Point-of-care haemoglobin testing in the TRACT trial

At the time the TRACT trial was conducted there were few options for reliable and validated technologies for the measurement of Hb, especially in the range of haemoglobins antipicated in children hospitalized with severe anaemia. We opted to use the HemoCue Hb 301 system (HemoCue AB, Angelholm, Sweden) which is a portable POC Hb testing kit, factory-calibrated against the reference method (without requirement for recalibration) and requiring virtually no maintenance. Disposable microcuvettes use microsamples of capillary or venous blood samples. The measurement range is 0 to 256 g/l, with results that are available in 10 s. Blood-based liquid controls were used for between-batch (microcuvettes) testing. It was selected based on its speed, reasonable cost and previous validation in relevant populations.^16,24^

## Point-of-care haemoglobin testing: was it reliable even in children with profound anaemia?

**Fig 1:**
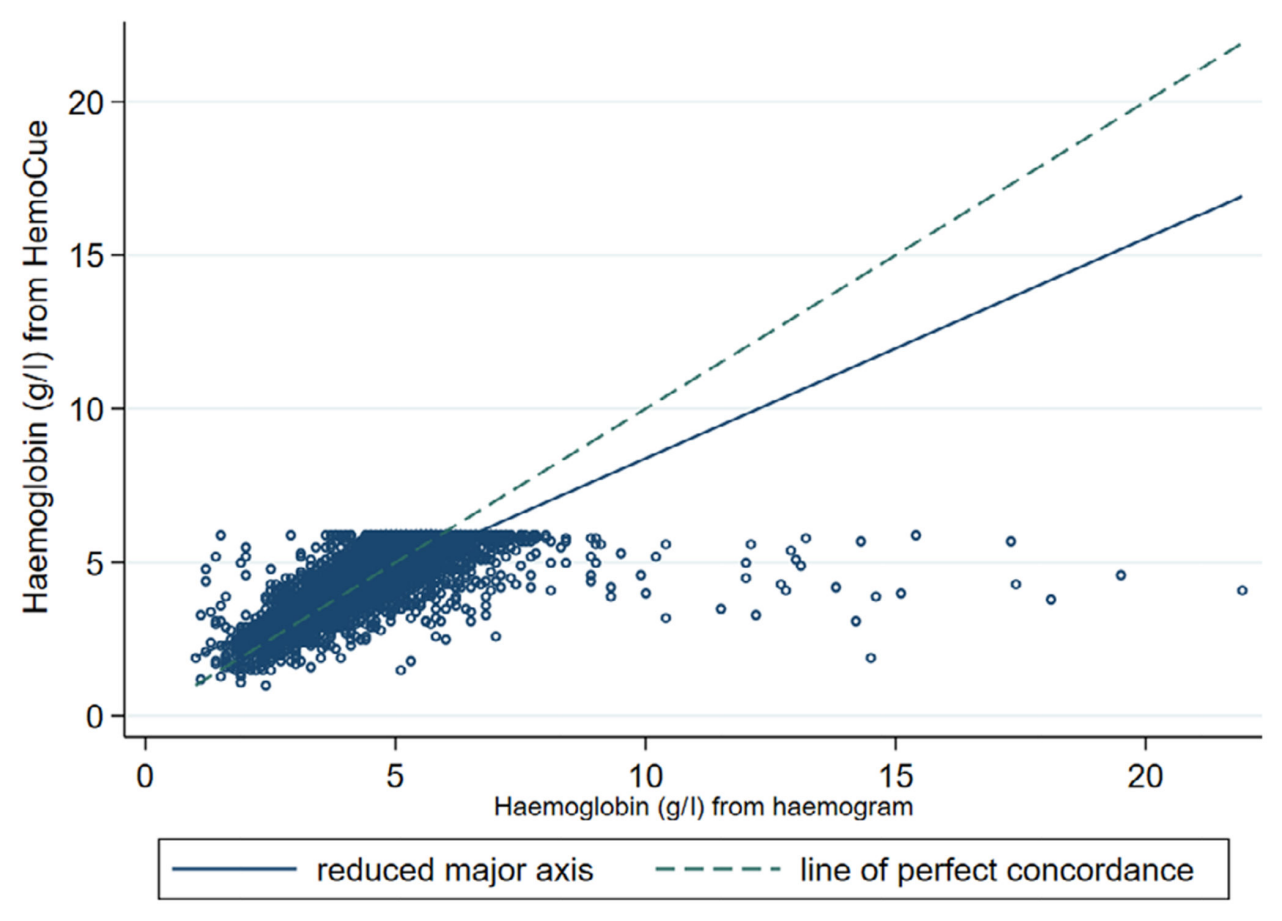
Comparison of haemoglobin measurements by Hemocue and haemogram. [Colour figure can be viewed at wileyonlinelibrary.com]

Whilst the HemoCue was used to obtain a haemoglobin at screening, baseline blood sampling included a full blood count (haemogram) which was taken straight after cassent/consent and before the start of any transfusion (per protocol). In 3 856/3 983 (97%) patients we were able to compare haemoglobin measurements using both of these methods (Fig 1). The median time from randomisation to the sample being taken was 9 min [interquartile range (IQR) 5–15 min]. Overall, 96% of haemograms were taken after randomisation and within 3 h, with 131 (3%) samples being taken before randomisation. For the analysis, we excluded haemogram-derived Hb taken >2 h from randomisation (*n* = 59) to ensure sampling was prior to starting any transfusion. The mean difference between Hb from HemoCue and Hb from a haemogram was very small (–0·046; *P* = 0·02 from a one-sample two-sided t-test) — a difference that is not clinically relevant. As such there was no indication of a systematic over- or underestimation of haemoglobin. The agreement between the two methods was compared by Lin’s concordance statistic [0·61 (95% CI 0·59–0·63)] and was fair. There were some outliers (26 had Hb levels >100 g/ l) which were due to errors in following the laboratory standard operating procedure (SOP), where blood had sedimented in samples that were not agitated before running the haemogram. In summary, we can be confident that POC Hb (HemoCue) performed well in a population with suspected severe anaemia and if implemented would result in savings across the health services if blood transfusion services required that a transfusion could only be ordered if supported by a Hb test.

## Alternative methods of estimating haemoglobin

### Haematocrit

Measurement of haematocrit count is easy, cheap and can be performed in most rural hospitals.

Haemoglobin is then generally estimated from haematocrit (Hct) using a conversion factor of Hct = 3 x Hb. Thus severe anaemia (Hb < 60 g/l) would equate to a Hct level of <18% and profound anaemia (Hb < 40 g/l) to a Hct level of <12%. We note that a number of investigators have reported that this conversion factor/estimation is not very accurate.^25,26^ This includes a community study conducted in healthy children in Tanzania and Mozambique demonstrating a prevalence of mild (80–110 g/l) and moderate anaemia (<80 g/l) which were 74% and 10%, respectively, when defined by Hb but the rates were lower if defined by Hct (42% and 3% respectively). This implies 72% (206/287) of the samples classified by Hb levels as moderate anaemia were not detected using Hct levels.^25^ Others have suggested using a lower conversion factor of 2·6;^27^ however, in practice this would be more difficult to calculate and impractical to inform immediate patient management by most health workers.

## Assessing the accuracy of haematocrit for identifying children with severe anaemia

**Fig 2:**
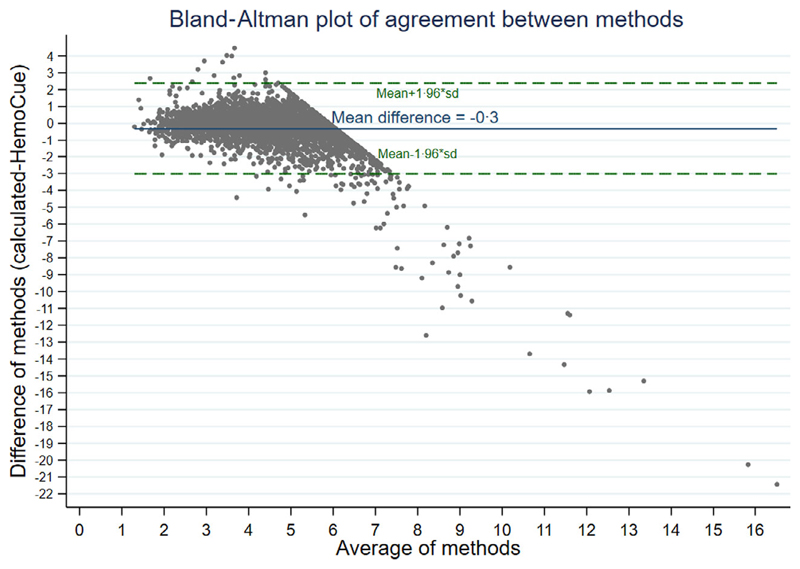
Comparison of haemoglobin estimated from haematocrit versus haemoglobin from HemoCue in children with severe anaemia. [Colour figure can be viewed at wileyonlinelibrary.com]

Haematocrit is a parameter included in a haemogram, so we were able to compare haemoglobin from the HemoCue to haemoglobin calculated from the haematocrit (using the conversion factor of HCT = 3 × Hb). The analysis was restricted to those with haematocrit measured from a haemogram taken within 2 h of randomisation [*n* = 3 855/3 983 (97%)]. This showed a poorer degree of agreement with a mean difference of –3 g/l between the two methods (*P* < 0·0001 one-sample two-sided *t*-test; Fig 2). Lin’s concordance coefficient was 0·54 (95% CI 0·52, 0·56) indicating the proxy estimation of Hb using haematocrit was worse than using Hb from a haemogram.

We also considered the specificity and sensitivity of using haematocrit cut-offs instead of HemoCue cut-offs for definitions of profound or severe anaemia, since these are often used by health services as ward-based tests. A 12% haematocrit cut-off is generally used to estimate profound anaemia (Hb < 40 g/l); we found this gave a sensitivity of 88% and specificity of 78%. An 18% haematocrit cut-off to estimate severe anaemia (Hb < 60 g/l) had a sensitivity of 83%. However, we were unable to calculate the specificity since all eligible children in the trial had a Hb < 60 g/l on the POC Hb result. In conclusion, using data from the TRACT trial, we showed that haematocrit provided a reasonable alternative as a method for detecting anaemia. In practice, however, there are other technical challenges in safe collection and processing of capillary blood often used for haematocrit assessment.^28^

**Fig 3:**
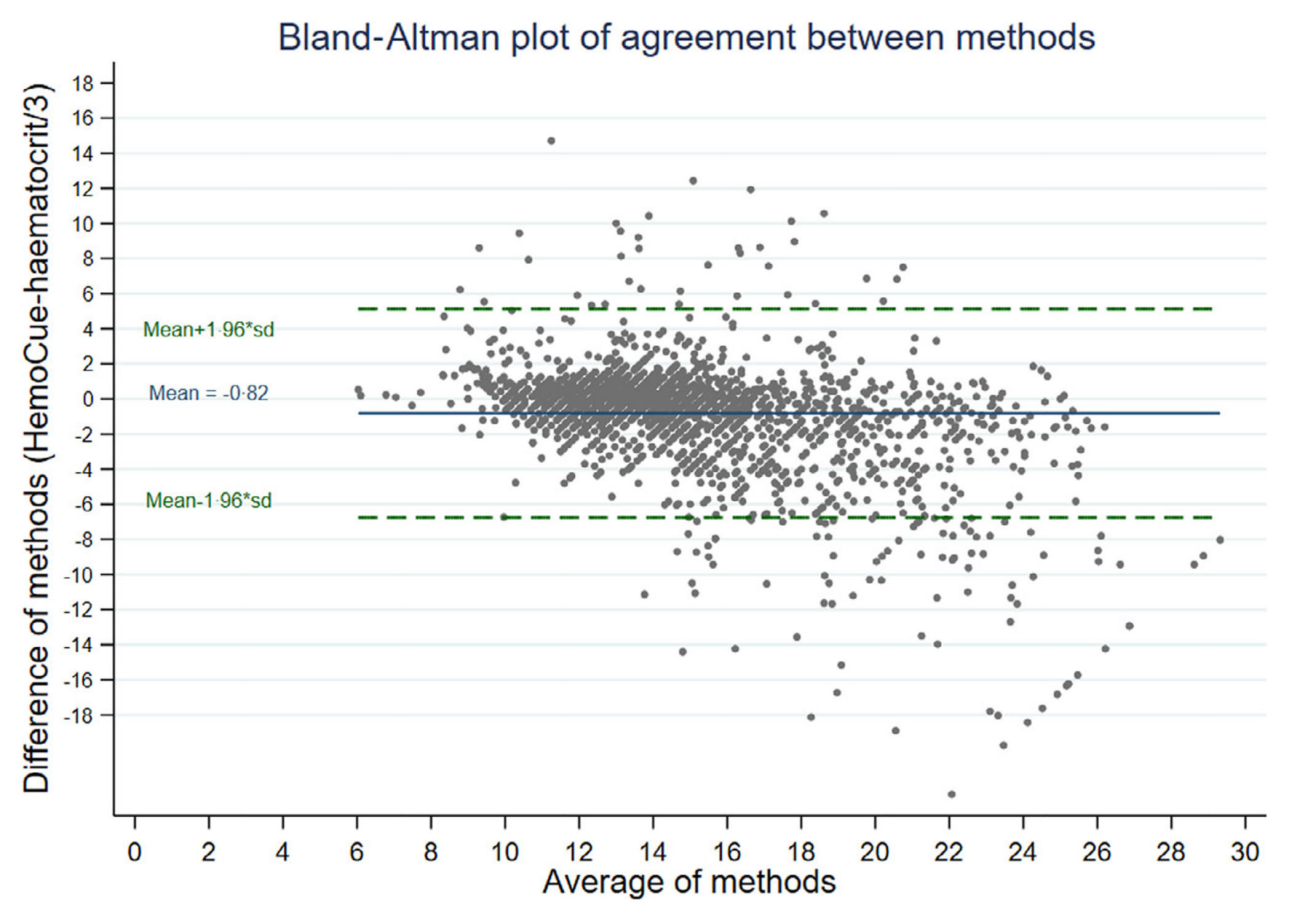
Haemoglobin estimated from haematocrit versus haemoglobin from HemoCue in Donor blood. [Colour figure can be viewed at wileyonlinelibrary.com]

Nevertheless, the comparison does not fully address whether ward-based tests, which in many hospitals include results from a haematocrit machine, may be less accurate than a haematocrit from a haemogram. In order for us to examine this specific question, we compared haemoglobin measured by HemoCue to estimated haemoglobin calculated from haematocrit (from a ward-based machine) taken from bags of donor whole blood used in the TRACT trial (n = 2 220 packs).^29^ The mean difference between the two methods was –0·82 (*P* < 0·0001, one-sample two-sided *t*-test; Fig 3) Lin’s concordance coefficient was 0·72 (95% CI 0·70–0·73). The good agreement between Hb calculated from Hct and Hb from the HemoCue is reassuring; however, the population did not include anyone with anaemia (who are generally screened out prior to blood donation) and there is wide variability (SD = 3·03). The better concordance for these tests in donor blood compared to haemogram and HemoCue in children with severe anaemia may also be simply due to the lack of a restriction so both methods on donor blood have a much wider range compared to the methods used for the children. Further research is needed to see if this method of Hb estimation could be introduced to or strengthened in paediatric wards to support best transfusion practice.

## WHO haemoglobin colour scale

Owing to the poor specificity of pallor for assessment of anaemia the WHO developed the haemoglobin colour scale (HCS) as an inexpensive, simple alternative for assessing anaemia. A systematic review examined its utility in a wide range of settings including 14 studies, most conducted in sub-Saharan Africa. The sensitivity of HCS was found to be high in most of the studies (75–97%) but the specificity was lower (41–98%) with both being higher for laboratory-based studies compared with more pragmatic ’real-life’ studies. It was concluded that where there is no access to laboratory a HCS may improve anaemia diagnosis; however further pragmatic research was recommended to help inform policy with a focus on ease of implementation.^30^ In Uganda, in children identified by health personnel (nurses and clinicians) as severely anaemic (based on assessment of pallor) for which they would empirically order a blood transfusion (if haemoglobin was unknown) the WHO Hemoglobin Colour Scale had a high specificity of 95·6 (90·7–98·4) but lacked sensitivity [43·1 (34·2–52·3)] for those with Hb < 50 g/l diagnosed using HemoCue.^14^

Relevant to this question, a study conducted in Malawi evaluated the characteristics of various manual Hb methods for accuracy, including assessment of the WHO HCS. The performance was judged against a reference standard (haemiglobincyanide method). For the HCS 95% of the results differ by only 48% below and 37% above the reference results compared to 6% and 16% for the HemoCue.^31^ In addition to examining performance of each haemoglobin method the researchers also compared a range of parameters including clinical usefulness, user friendliness, speed, training time and economic costs for six haemoglobin measurement methods. The authors proposed that these could be applied as a practical model for gathering evidence about test efficiency that could be adapted for use in other resource-poor settings. On this basis, HemoCue was found to be the ideal in terms of ease of use, speed, minimal requirement for training and reliability of the results; however it was the most expensive (0·75 US dollars/test). The Ugandan study, conducted a decade later, found the cost of the HemoCue test had increased (~4·0 USD/test at 2019 prices, after an initial cost of between 250 and 350 USD for the HemoCue analyser). This was more costly than then next best test, Sahli’s method (0·25 USD/test, after an initial cost of 40–50 USD for the meter); however, Sahli’s Hb estimation requires a functioning and accessible laboratory service^14^ and it is less practicable for emergencies and for ‘out of hours’ admissions.

## TRACT trial management algorithm

To update current guidelines, with the new findings from the TRACT trial, a consensus severe anaemia management algorithm was developed with the aim of improving evidence-based clinical practice and averting the unnecessary use of transfusion in children with uncomplicated severe anaemia.^32^ However, two vital components of the transfusion management algorithm included both clinical and Hb monitoring. Using data from the TRACT trial, we were able to show that a minimum of three additional measurements of Hb over 48 h of hospital stay would identify ~50% developing severe and complicated anaemia since 76% of children required a transfusion following of a drop in Hb < 40 g/l. From this watch and wait policy there was no evidence of short- or long-term adverse events (morbidity and mortality). The biggest concern raised at the meeting was that future evidence-based practice would require the provision of POC Hb testing — something that most hospitals do not have.

## Role of point-of-care testing in patient management

Point-of-care (POC) testing is a diagnostic procedure done at or near the site of care and has the benefit of providing immediate results to a clinician (and patient where necessary) without having to wait hours or even days for sample transport and laboratory processing. Practical considerations for the POC products are that they do not rely on physical infrastructure (including electricity, refrigeration, and/or running water or all). Increasingly, POC technology is being introduced to doctors’ clinics to meet specific patients’ indications in developed countries.^33^ These types of diagnostic platforms could also enable better approaches to low-resource-setting tests. To date, POC tests used in resource-limited settings have principally focused on infectious diseases that need prompt diagnosis and treatment, such as HIV, tuberculosis and malaria.

## Point-of-care haemoglobin testing : using the TRACT trial as an exemplar

The development and design of clinically and commercially viable POC products for low- and middle-income countries (LIMCs) in a reasonable time and clinical studies that show implementation results with better patient outcomes. The latter consideration has often been difficult to prove, since implementation studies with survival as an outcome are rare. In the TRACT trial, POC Hb was incorporated as a screening procedure to identify children with severe anaemia and to inform use of emergency interventions (in this case transfusion), thus demonstrating its clinical utility. For emergency interventions, this aspect is critical since it can be done at any time of the day/night, during holidays and when there is no power — all of which reduce the availability and speed of laboratory-based diagnoses. Whilst mortality rates in children with uncomplicated anaemia were too low either to demonstrate or refute any benefits of an immediate transfusion compared to control (WHO standard of care), the large multicentre TRACT trial demonstrated that in children with uncomplicated severe anaemia a triggered transfusion strategy was safe and cost-effective but required clinical and Hb monitoring.

## Other patient groups where point-of-care haemoglobin testing could be useful in African hospitals

Severe anaemia, requiring transfusion, is a very common maternity-related complication.^34^ Quite often moderate to severe anaemia arises during pregnancy, but is more critically in the peripartum period, where between 20 and 30% of maternal deaths in Africa are due to postpartum haemorrhage.^35^ Lack of access to blood transfusions and poorly organised health services have been implicated in the high maternal mortality rates in African countries. Point-of care Hb at busy antenatal clinics to identify and treat anaemia and in the obstetric units would facilitate improved management since the degree of blood loss in postpartum haemorrhage is often underestimated.

Other emergencies which would benefit from rapid diagnosis of haemoglobin include the growing burden of trauma, largely from road traffic accidents, on the continent.^36^

## Barriers to adoption

A number of barriers currently prevent the adoption of existing, and the introduction of new diagnostics in LMICs due, in part, to challenges posed by centralisation of laboratory systems. Whilst centralised laboratories play an important role in providing medical diagnostic services, they require significant infrastructure and trained personnel. The aim of POC tests is to supplement, not replace, centralised laboratory testing, thus bringing diagnostics to the hospital ward or bedside, enabling timely diagnosis and leading to expedited clinical decisions. In the case of severe anaemia for comprehensive clinical care, we recognised that an initial POC Hb should not replace the requirement for essential laboratory investigations (including full blood count or haemogram) to refine diagnosis and guide comprehensive patient management. To avoid a one size fits all scenario, the assessment process of any diagnostic test should take place within a specific clinical indication and with a specified purpose for the diagnostic test. Drain and colleagues proposed a framework for the assessment of point-of-care tests, and how their efficacy can be defined and evaluated in operational research that is centred on clinical decision-making.^37^ They refer to the WHO ASSURED criteria which outline the ideal characteristics of a POC for resource-limited settings.

Originally developed as a benchmark for clinical tests to identify sexually transmitted disease, they consist of the following criteria: Affordable, Sensitive (low number of false negatives), Specific (low numbers of false positives), User-friendly (and minimal training required), Rapid and robust (i.e. does not need refrigeration), Equipment-free and Deliverable to those who need it.^38^ These criteria can equally be applied to POC Hb, where we believe there is a strong case for advocating for the introduction of new POC Hb diagnostics if supported by well executed studies on diagnostic accuracy, clinical effect and cost in a non-clinical trial context. Most published studies of point-of-care tests in resource-limited settings have focused on analytical performance, with few studies assessing a test’s effects on patient-centred outcomes. Thus, assessing the impact of using POC Hb in routine care on patient-focused outcomes (e.g., survival) is vitally important.

## Conclusions

We advocate for POC Hb to be more widely available for hospitals across sub-Saharan Africa, so that requests for blood transfusion are better supported by prompt, accurate and serial Hb testing. This could have dual benefits, so that severe and life-threatening anaemia is identified rapidly, and cost-savings to the blood transfusion services realised, reducing the number of unwarranted prescriptions of blood for transfusions.
